# Wolff‐Parkinson‐White Mimicking Posterior STEMI: When Even AI Gets It Wrong

**DOI:** 10.1002/ccr3.70363

**Published:** 2025-03-24

**Authors:** Marco Biasin, Lorenzo Bonadiman, Francesca Vassanelli, Flavio Ribichini

**Affiliations:** ^1^ Division of Cardiology University of Verona Verona Italy

**Keywords:** accessory pathway, acute coronary syndrome, artificial intelligence, Wolff‐Parkinson‐white

## Abstract

Wolff‐Parkinson‐White (WPW) can mimic ischemic ECG patterns, leading to potential misdiagnosis of acute coronary syndromes. This case highlights the importance of integrating clinical evaluation, echocardiography, and troponin testing with AI‐assisted ECG analysis to accurately differentiate pre‐excitation from true ischemia and avoid inappropriate interventions.

## 1 Introduction

Wolff‐Parkinson‐White (WPW) is a congenital cardiac conduction disorder characterized by the presence of an accessory pathway, known as the bundle of Kent, which allows electrical impulses to bypass the normal atrioventricular (AV) node. This anomalous conduction pathway can lead to premature ventricular depolarization and predisposes patients to potentially life‐threatening arrhythmias, including atrioventricular reentrant tachycardia and, less commonly, atrial fibrillation degenerating into ventricular fibrillation. The underlying etiology of WPW is primarily congenital, resulting from the presence of aberrant muscle bundles, known as accessory pathways (APs), that connect the atrium to the ventricle outside the regular atrioventricular (AV) conduction system. These APs are embryologic remnants caused by incomplete development of the AV annuli, leading to a lack of complete separation between the atria and ventricles during embryogenesis. This congenital anomaly is present from birth, although clinical manifestations can occur at any age [1].

This condition presents significant diagnostic challenges, particularly in emergency settings where patients may exhibit symptoms such as chest pain. WPW is diagnosed through characteristic electrocardiographic (ECG) findings, including a shortened PR interval (< 0.12 s), prolonged QRS complex (> 0.12 s), and the presence of a delta wave, a slurred, slow‐rising onset of the QRS complex. The degree of pre‐excitation can vary, depending on the location of the accessory pathway (AP) and the conduction properties of the AV node. Pre‐excitation on the surface ECG can be intermittent and may even disappear in some cases [1]. These ECG changes can mimic ischemic alterations (Table 1), such as ST‐segment depression or T‐wave inversion, leading to diagnostic uncertainty, particularly in emergency settings where patients may present with chest pain [2, 3].

**TABLE 1:** ECG Differences between WPW and STEMI.

ECG feature	WPW syndrome	STEMI
PR interval	Shortened (< 120 ms) due to pre‐excitation	Normal or prolonged (if AV block is present)
Delta wave	Present (slurred upstroke in QRS, short PR interval, widened QRS)	Absent
QRS complex morphology	Widened QRS complex due to ventricular pre‐excitation	Generally normal or slightly widened if ischemic conduction delay is present
ST segment elevation	Possible but typically discordant with the QRS complex	Concave or convex upward ST‐segment elevation in at least two contiguous leads
Location of ST changes	Diffuse and variable, depending on the location of the accessory pathway	Localized to specific coronary artery territories (e.g., inferior leads for RCA occlusion)
Reciprocal changes	Absent or atypical	Present in anatomically opposite leads

In recent years, artificial intelligence‐based tools for ECG interpretation have been integrated into clinical practice, offering the potential to improve diagnostic accuracy and assist clinicians in emergency settings. However, these tools have yet to be extensively tested across all clinical scenarios, such as in cases of Wolff‐Parkinson‐White, where ECG patterns may present unique challenges.

## 2 Case History

**FIGURE 1:**
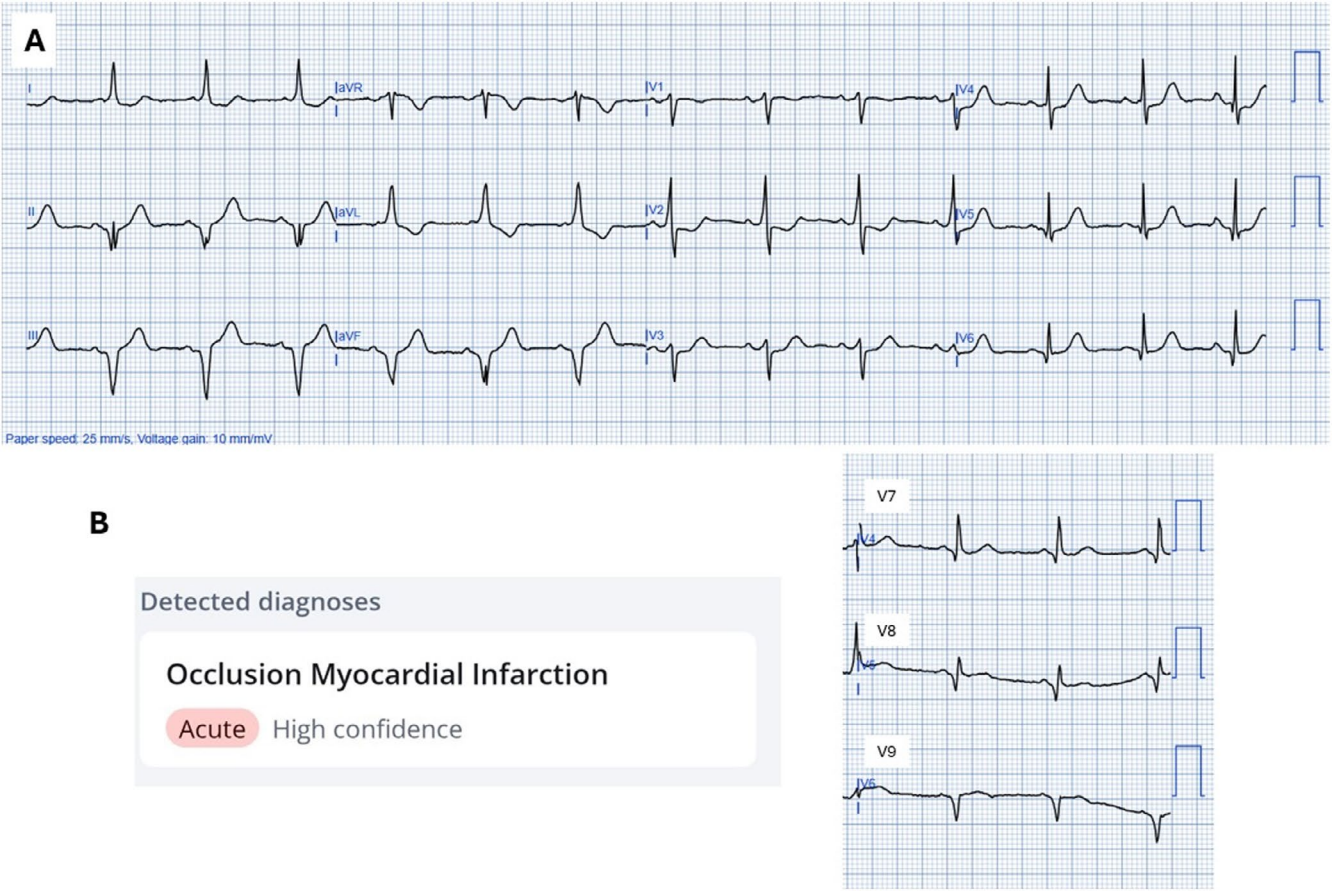
Initial ECG: (A) ST‐segment depression in V1–V3; (B) Posterior leads showing ST‐segment elevation with AI interpretation.

An 83‐year‐old woman with a history of hypertension and hyperlipidemia presented with sudden‐onset retrosternal chest pain accompanied by dyspnea. On arrival, paramedics performed an ECG showing ST‐segment depression in leads V1–V3, raising suspicion for posterior STEMI (Figure 1A). Posterior leads (V7–V9) were applied, revealing ST‐segment elevation of at least 0.5 mm, further supporting the diagnosis of acute posterior myocardial infarction (Figure 1B). An AI‐assisted ECG tool (PMcardio OMI AI ECG model, Queen of Hearts, Powerful Medical, Slovakia) interpreted the findings as consistent with acute occlusive myocardial infarction. Based on these results and the patient's clinical presentation, the decision was made to transfer her urgently to the emergency department for further evaluation.

## 3 Differential Diagnosis, Investigations, and Treatment

**FIGURE 2:**
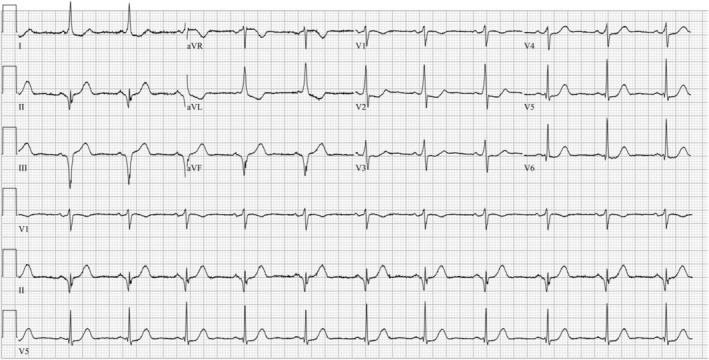
ECG in the emergency department showed pre‐excitation.

At the emergency department, a repeat ECG confirmed the pre‐hospital ST‐segment abnormalities but also revealed features of pre‐excitation consistent with WPW, including a delta wave and a shortened PR interval (Figure 2). This finding provided an alternative explanation for the observed ST‐segment changes. Bedside echocardiography demonstrated preserved left ventricular function with no segmental wall motion abnormalities. Serial high‐sensitivity troponin assays were within normal limits, effectively ruling out acute coronary syndrome. The observed ECG changes were attributed to WPW, which can mimic ischemic patterns, including both ST‐segment depressions and elevations, in the presence of pre‐excitation. As the patient was hemodynamically stable and her chest pain resolved without intervention, she was admitted for short‐term cardiac monitoring.

## 4 Conclusion and Results

The patient was observed for 24 h without recurrence of symptoms or arrhythmias and was subsequently discharged with a recommendation for outpatient follow‐up. An electrophysiological evaluation was scheduled to assess the accessory pathway and discuss potential ablation therapy.

## 5 Discussion

The WPW pattern is characterized by an accessory conduction pathway that leads to pre‐excitation of the ventricles, visible as a delta wave on the ECG. This pre‐excitation can complicate ECG interpretation, particularly in patients with chest pain, as it mimics ischemic changes such as ST‐segment abnormalities. The abnormal activation and repolarization caused by the accessory pathway often result in ST‐T wave alterations, which can be misinterpreted as ischemic changes. Consequently, the presence of WPW can complicate the diagnosis of ischemia, infarction, hypertrophy, and pericarditis, as these conditions may not be reliably distinguished in the context of the WPW pattern [4]. In this case, the patient's ECG showed ST‐segment depression in the anterior precordial leads (V1–V3), raising suspicion of a posterior STEMI. The use of posterior leads, which revealed ST‐segment elevation, further supported the possibility of an acute coronary event, prompting cardiology consultation and the use of AI‐assisted diagnostic tools. The delta wave and resulting repolarization abnormalities in WPW can distort the ECG, increasing the risk of mistaking benign changes for acute coronary syndromes in patients presenting with chest pain [2, 3].

Management of WPW differs significantly from that of true STEMI. In STEMI, immediate reperfusion therapy, typically with primary percutaneous coronary intervention or fibrinolysis, is the cornerstone of treatment. Conversely, in WPW without true ischemia, invasive coronary interventions are unnecessary and could expose the patient to unwarranted risks. WPW management focuses on controlling arrhythmias and preventing sudden cardiac death. In this case, a 24‐h ECG monitoring was performed in the Emergency Department to rule out arrhythmias that could have explained the chest pain. A cardiac event monitor, worn for 24–48 h, is effective in detecting abnormal heart rates, electrical impulse patterns, and pre‐excitation. It also helps assess the risk profile, as patients with multiple accessory pathways are at a higher risk of sudden cardiac death. Conversely, intermittent pre‐excitation, characterized by the intermittent disappearance of delta waves even at normal heart rates, suggests a lower risk profile. In this patient, continuous monitoring showed no evidence of high‐risk arrhythmias, supporting the decision to avoid unnecessary invasive interventions [5].

In this case, the AI‐assisted ECG tool suggested a possible occlusive myocardial infarction based on the initial findings. AI‐based ECG interpretation tools have shown promise in enhancing diagnostic accuracy, particularly by identifying subtle ischemic changes that might be missed by human readers. At our institution, a new and advanced AI tool, the PMcardio OMI AI ECG model (Queen of Hearts, Powerful Medical, Slovakia), is available. This model has demonstrated superior performance compared to conventional STEMI criteria and is comparable to interpretations by specialized ECG experts in detecting invasively confirmed acute coronary occlusion. It achieves high accuracy and consistent performance across various demographic, electrocardiographic, and infarct territory subgroups [6]. However, it is important to recognize the limitations of AI tools, especially in cases of pre‐existing conditions like WPW, which can interfere with standard ECG interpretation algorithms. While AI can assist in recognizing ischemic patterns, its diagnostic capabilities are not infallible and should be used alongside clinical evaluation and additional diagnostic modalities, as demonstrated in this case [7].

In addition to the challenges posed by ECG interpretation, this case underscores the importance of integrating other diagnostic tools into the clinical workflow when evaluating patients with chest pain. Bedside echocardiography, for instance, provided crucial information by demonstrating normal left ventricular function and the absence of regional wall motion abnormalities, effectively ruling out myocardial infarction. This finding, combined with normal troponin levels, was essential in excluding acute coronary syndrome and redirecting attention to pre‐excitation as the likely cause of the ECG changes [8]. In cases where initial investigations point toward a life‐threatening diagnosis such as STEMI but further evaluation reveals an alternative explanation, it is essential for clinicians to maintain a broad differential diagnosis and avoid anchoring bias.

This case highlights the diagnostic challenges posed by Wolff‐Parkinson‐White (WPW) syndrome when presenting with chest pain, particularly due to its potential to mimic ischemic ECG patterns, such as those observed in posterior myocardial infarction. It underscores the necessity of integrating clinical judgment, bedside echocardiography, and serial troponin testing with AI‐assisted ECG analysis to accurately distinguish between true ischemia and pre‐excitation‐related changes. Additionally, this case illustrates the limitations of AI in interpreting ECGs in the presence of conduction abnormalities, reinforcing the need for complementary diagnostic tools to avoid misdiagnosis and ensure accurate patient management.

## Author Contributions

**Marco Biasin:** conceptualization, investigation, writing – original draft, writing – review and editing. **Lorenzo Bonadiman:** conceptualization, writing – original draft. **Francesca Vassanelli:** writing – review and editing. **Flavio Ribichini:** writing – review and editing.

## Consent

Written informed consent was obtained from the patient.

## Conflicts of Interest

The authors declare no conflicts of interest.

## Data Availability

The data that support the findings of this study are available on request from the corresponding author. The data are not publicly available due to privacy or ethical restrictions.
